# Pediatric Simplified Acute Physiology Score II: Establishment of a New, Repeatable Pediatric Mortality Risk Assessment Score

**DOI:** 10.3389/fped.2021.757822

**Published:** 2021-10-28

**Authors:** Stefan Irschik, Jelena Veljkovic, Johann Golej, Gerald Schlager, Jennifer B. Brandt, Christoph Krall, Michael Hermon

**Affiliations:** ^1^Division of Neonatology, Pediatric Intensive Care and Neuropediatrics, Medical University of Vienna, Vienna, Austria; ^2^Medical University of Vienna, Vienna, Austria; ^3^Center for Medical Statistics, Informatics and Intelligent Systems, Medical University of Vienna, Vienna, Austria

**Keywords:** critical care, mortality, pediatrics–children, risk assessment, scoring systems

## Abstract

**Objectives:** In critical care it is crucial to appropriately assess the risk of mortality for each patient. This is especially relevant in pediatrics, with its need for accurate and repeatable scoring. Aim of this study was to evaluate an age-adapted version of the expanded Simplified Acute Physiology Score II; (p-SAPS II), a repeatable, newly-designed scoring system compared to established scores (Pediatric Sequential Organ Failure Assessment Score/pSOFA, Pediatric Logistic Organ Dysfunction Score-2/PELOD-2 and Pediatric Index of Mortality 3/PIM3).

**Design:** This retrospective cohort pilot study included data collected from patients admitted to the Pediatric Intensive Care Unit (PICU) at the Medical University of Vienna between July 2017 through December 2018.

**Patients:** 231 admissions were included, comprising neonates (gestational age of ≥ 37 weeks) and patients up to 18 years of age with a PICU stay longer than 48 h.

**Main Outcomes:** Mortality risk prediction and discrimination between survivors and non-survivors were the main outcomes of this study. The primary statistical methods for evaluating the performance of each score were the area under the receiver operating characteristic curve (AUROC) and goodness-of-fit test.

**Results:** Highest AUROC curve was calculated for p-SAPS II (AUC = 0.86; 95% CI: 0.77–0.96; *p* < 0.001). This was significantly higher than the AUROCs of PELOD-2/pSOFA but not of PIM3. However, in a logistic regression model including p-SAPS II and PIM3 as covariates, p-SAPS II had a significant effect on the accuracy of prediction (*p* = 0.003). Nevertheless, according to the goodness-of-fit test for p-SAPS II and PIM3, p-SAPS II overestimated the number of deaths, whereas PIM3 showed acceptable estimations. Repeatability testing showed increasing AUROC values for p-SAPS II throughout the clinical stay (0.96 at day 28) but still no significant difference to PIM 3. The prediction accuracy, although improved over the days and even exceeded PIM 3.

**Conclusions:** The newly-created p-SAPS II performed better than the established PIM3 in terms of discriminating between survivors and non-survivors. Furthermore, p-SAPS II can be assessed repeatably throughout a patient's PICU stay what improves mortality prediction. However, there is still a need to optimize calibration of the score to accurately predict mortality sooner throughout the clinical stay.

## Introduction

One of the major challenges Intensive Care Units (ICUs) face is to objectively evaluate a patient's condition in cases of critical illness to derive a predictive outcome. Before the first scoring system was developed, assessments were highly subjective (1).

Assessing a patient's mortality risk at time of admission is of highest priority in clinical routines. While death rates are usually low in pediatric intensive care (PICU) patients, especially when compared to their adult counterparts (2), a scoring method useable across all situations–and for every patient–is nevertheless needed to produce accurate predictions of high-mortality risk patients. The ability to monitor a patient's clinical course over time, using repeated assessments throughout the PICU stay, is another area of high relevance (3, 4).

In clinical practice, there are already different scoring methods available. Attempts have been made to create new scores or update and adapt existing scores for use in pediatrics, such as Pediatric Index of Mortality (PIM) (5–7), Pediatric/Age-adapted Sequential Organ Function Assessment Score (pSofa/age-adapted SOFA) (8, 9) or the Pediatric Logistic Organ Dysfunction Score (PELOD) (2, 10). Furthermore, scores for adults have been adapted for use in children by utilizing cut-offs from existing scores or appropriate age-adapted norm values and percentiles, such as the pSOFA/age-adapted SOFA (8, 9). However, some aspects should be taken into consideration when applying these scores. PIM3, for example, is well-established and produces accurate results. Its use, however, is limited to the first assessment within the 1 h after admission (5). PELOD was designed for children with multiple organ dysfunction syndrome (2, 10), while pSOFA assesses the severity of organ dysfunction in the framework of sepsis (8, 9).

Due to varying aspects of the aforementioned scores, we sought to develop a scoring model that would meet the requirements of PICUs in the most accurate way possible. An ideal scoring model should have a high discrimination potential for predicting mortality risk in both high- and low-mortality risk children. The scoring model should be applicable and repeatable in every situation to possibly assess treatment success throughout the patient's course.

We therefore developed an age-adapted version of the expanded Simplified Acute Physiology Score II (expanded SAPS II) (11) for use in children. To our knowledge, no such version has been published to date. The decision to adapt the expanded SAPS II rather than SAPS II (12) or Simplified Acute Physiology Score 3 (SAPS 3) (13, 14) was that SAPS II has been shown to overpredict mortality, while SAPS 3 must be customized before it can be effectively used in clinical routines (11, 15).

## Questions and Objectives

The objective of this study was to compare the performance of our newly-created, age-adapted and modified expanded SAPS II, or pediatric SAPS II (p-SAPS II), with PIM3, PELOD-2 and pSOFA. The primary endpoints were the prediction of mortality risk within the1 day of the PICU stay and the general discrimination potential between survivors and non-survivors.

## Methods

The presented study was performed as a retrospective cohort pilot study.

The study protocol was submitted to the Ethic Committee of the Medical University of Vienna. A positive vote was achieved in March 2019 (EK number 1142/2019).

An age-adapted variant of expanded SAPS II (11) was generated using a three-step approach. First, we adapted the metrical parameters from the expanded SAPS-II according to age-adapted norm values and percentiles. Moreover, five age-categories were defined, similar to the original SAPS II under the assumption that the younger a child is, the higher the mortality risk would be in the framework of a critical illness.

In a second step, the variable “chronic diagnoses” which was seen as not applicable for the pediatric population was exchanged with the variable “main pediatric diagnosis” specific for the pediatric setting (Supplementary Material Table 1). The new variable strongly orientates on the diagnosis catalog and classification used in PIM 3 (5). Delphi method performed by the authors was used to expand the list of important diagnoses according to our standard patient population. Since this study was performed at a PICU, which is part of the national pediatric heart center many of the diagnoses were of cardiac origin. To classify diagnoses according to the risk categories, an in-depth literature research was performed using MEDLINE. Appropriate studies were searched to categorize each diagnosis according to relative mortality risk. Primarily, studies offering odds ratios relating to the diagnoses were preferred, but calculated odds ratios were not always available. For this reason, we chose the risk of mortality, because it was always presented. As in Straney et al. (5), the diagnoses and reasons for admission were grouped into three categories: low-risk (odds ratio lower 1), high-risk (odds ratio between 1 and 5) and very high-risk (odds ratio higher 5). When odds ratios were unavailable, the published mortality risks were used for comparison (Supplementary Material Table 1).

Relative risk was graded in three categories: “Low-risk diagnoses with a typical risk of mortality lower than 4 or 5%,” “High-risk diagnoses with a risk of mortality between 4 or 5% and 20 or 30%,” and “Very high-risk diagnoses: Risk of mortality higher than 20 or 30%.”

The total sum of points, normally given to the “chronic disease” category, were newly distributed to the variable “pediatric diagnosis” according to the risk assessment grades (Table 1).

**Table 1:** Pediatric diagnoses and new distribution of points according to the mortality risk.

Low-risk	9 Points (original version of SAPS II)	= >	2 Points (age-adapted version of SAPS II)
High-risk	13 Points (original version of SAPS II)	= >	8 Points (age-adapted version of SAPS II)
Very high-risk	17 Points (original version of SAPS II)	= >	29 Points (age-adapted version of SAPS II)

### The Study Population

All patients admitted to the PICU between July 2017 and December 2018 were extracted from the patient data management system (PDMS), Philips Healthcare, InteliSpace Critical Care and Anesthesia, Version J.00.00, country of origin: Netherlands (16).

All consecutive PICU admissions comprising neonates (gestational age of ≥ 37 weeks) and patients up to 18 years were included, irrespective of whether a child was admitted more than once during the observation period. The exclusion criteria were: premature (<37 weeks of gestation) at the time of admission; pregnancy; length of PICU stay for <48 h (only applicable to survivors); transfer to another PICU (except to the neonatal ICU of our hospital), and children admitted to the PICU that were not intensive care patients.

Altogether, there were 508 admissions to the PICU during this time period, of which 231 could be included according to our criteria.

### Data Management and Statistical Methods

As PIM 3, per definition, is only to be assessed directly at admission and because we wanted to compare the performance of the different scores directly with each other, scores were created primarily with respect to Day 1 (admission day); only the first 4 h were considered. The time point for score assessment was the same for every patient and every score so direct comparability of the predictive potential at admission to the PICU was insured. Due to the retrospective character of the study it was necessary that all variables needed for calculating the scores were part of routine monitoring, as they had already been measured. If a parameter was missing or not stated pathological it was considered missing and not taken into consideration for calculation of the score. If a variable had multiple values, the most abnormal value was used for scoring to assess the patient's most critical situation. Descriptive statistic was used to classify the study population. The correlation between the scores and mortality was analyzed by binary logistic regression and the chi-squared test. Repeatability testing of p-SAPS II was performed *via* daily p-SAPS II assessment compared to the corresponding PIM 3. The main statistical methods to evaluate each score's performance; namely, the discrimination potential between death and survival and the accuracy of predictions of each score, were the area under the receiver operating characteristic curve (AUROC) and goodness-of-fit test. The differences of AUROC curves were tested for significance by using the program function “ROC Analysis” of IBM SPSS statistics (versions 25.0.0.0 and 26.0.0.0, IBM corporation in the USA and other countries) for statistical evaluation. The goodness-of-fit test was performed using the chi-Square Goodness of Fit Test Calculator. (2020, December 13) from “https://stats.libretexts.org/@go/page/8624” (17).

## Results

A total of 231 admissions (196 patients) were included in the study. A detailed description of the demographic data can be seen in Table 2. Owed to our national pediatric heart center he most prevalent reasons for PICU admissions were cardiovascular disorders (132/231; 57.1%) followed by respiratory-related cases (55/231; 23.8%). Admissions concerning other organ systems were less frequent. Children with congenital diseases had the highest admission frequency (146/231; 63.2%), with congenital heart disease being the primary reason with 127 admissions (127/231; 55%).

**Table 2:** Descriptive statistic of the study population. In the study some patients were included more than one time as each admission was assessed.

	***N (%)***
**Patients**	196 (100)
Female	91 (46.4)
Male	105 (53.6)
**Age (months)**	
Newborn (0–30 days)	69 (35.2)
Infant (1–12 months)	58 (29.6)
Toddler, preschool child (1–5 years)	41 (20.9)
School child (6–12 years)	18 (9.2)
Adolescent (12–18 years)	10 (5.1)
**Preterm birth in the patient's history (age** **>** **37 weeks at the time of admission) Number of admissions per patient (during the observation period)**	22 (11.2)
1 stay	167 (85.2)
2 stays	24 (12.2)
3 stays	4 (2.0)
4 stays	1 (0.5)
**Primary reason for PICU admission**	**231 (100)**
Respiratory	55 (23.8)
Neurological	25 (10.8)
Cardiovascular	132 (57.1)
Hepatic	1 (0.4)
Genitourinary	1 (0.4)
Gastrointestinal	2 (0.9)
Musculoskeletal	1 (0.4)
Undetermined	6 (2.6)
Mixed	7 (3.0)
Metabolic	1 (0.4)
**Non-survivors**	**15(7.6)**
Female	2 (2.1)
Male	13 (12.4)
Newborn (0–30 days)	5 (7.2)
Infant (1–12 months)	4 (6.9)
Toddler, preschool child (1–5 years)	3 (7.3)
School child (6–2 years)	1 (5.6)
Adolescent (12–18 years)	2 (20)
**Primary reason for PICU admission/ Non-survivors**	
Respiratory	8
Cardiovascular	4
Undetermined	1
Mixed	1
Metabolic	1

*Mortality by number of patients is depicted with 7.6% whereas mortality after admission is 6.4%*.

Within the observed time period, 15 patients (15/196; 7.6%) out of the 231 admissions (15/231 admissions; 6.5%) died during the PICU stay.

### ROC Results

**Figure 1:**
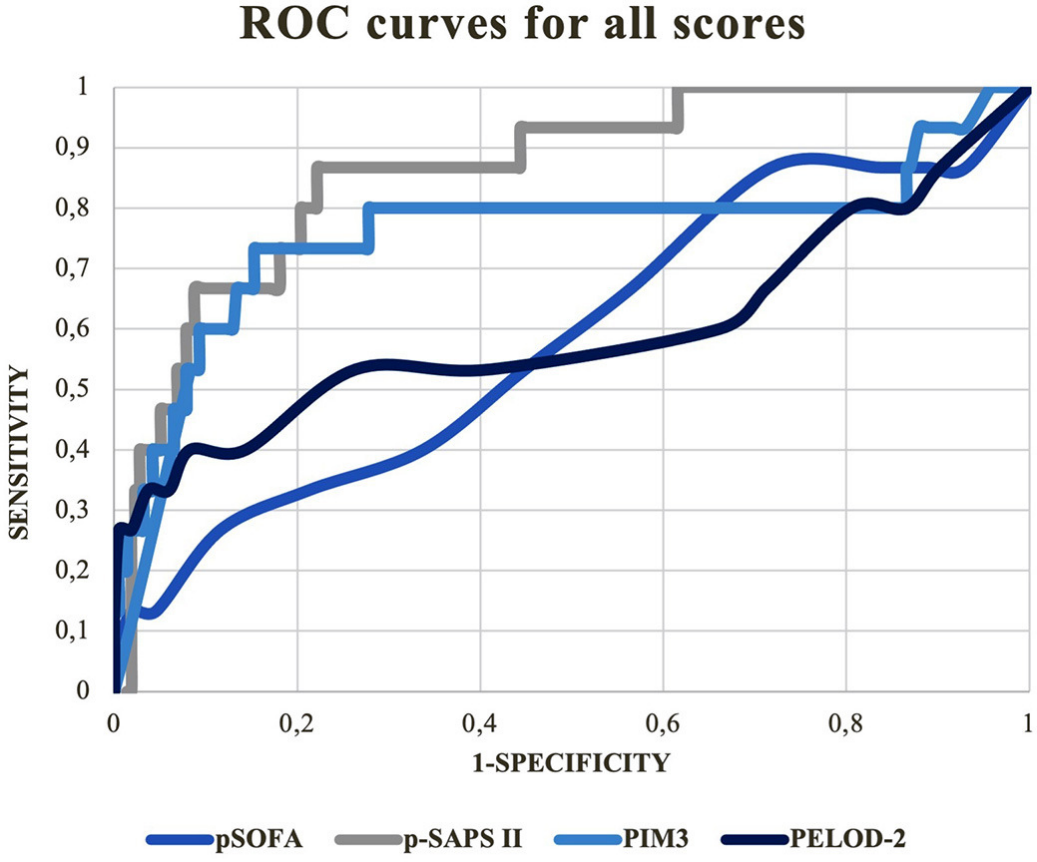
ROC curves for the tested mortality scores.

Receiver operation characteristic (ROC) curves for all scores are depicted in Figure 1.

**P-SAPS II:** AUC = 0.86; 95% CI: 0.77–0.95; *p* < 0.001.

**PELOD-2:** AUC = 0.60; 95% CI: 0.40–0.80; *p* = 0.102.

**PIM3:** AUC = 0.76; 95 % CI: 0.58–0.94; *p* = 0.004.

**pSOFA:** AUC = 0.58; 95% CI: 0.42–0.75; *p* = 0.329.

According to AUROC comparison, significant differences were only seen between p-SAPS II and PELOD-2: (AUROC difference =-0.26; *p* = 0.003) and p-SAPS II and pSOFA: (AUROC difference = −0.28; *p* = 0.001). Because the AUROC difference between p-SAPS II and PIM3 was not significant (AUROC difference = −0.1; *p* = 0.11) a logistic regression was performed to find possible significant differences between p-SAPS II and PIM3 as shown below. The AUROC of PIM3 did not significantly differ from the AUROCs of PELOD-2 and pSOFA.

### Goodness-of-fit Test for p-SAPS II and PIM3

Table 3 demonstrates the goodness-of-fit tests for p-SAPS II and PIM3 which is relevant to assess the predictive potential of a score. According to calculated *p*-values, the differences between the observed and expected data were not statistically significant for PIM3 (*p* = 0.774), in contrast to p-SAPS II (*p* < 0.001). The ratio of “observed” against “expected” deaths (O/E ratio) for p-SAPS II was 0.17, and 0.69 with PIM3, meaning that p-SAPS II overpredicts mortality.

**Table 3:** Goodness of fit test for p-SAPS II and PIM3 respectively.

**Mortality risk**	**p-SAPS II (*n =* 231)**				**PIM3(*n =* 231)**			
	**χ^2^ = 62.433, *df* = 9, *p <* 0.001**				**χ^2^ = 4.049, *df* = 7, *p =* 0.774**			
	**Died**		**Survived**		**Died**		**Survived**	
	**O**	**E**	**O**	**E**	**O**	**E**	**O**	**E**
0–10%	0	1.1	22	20.9	6	9.95	193	189.05
>10–20%	0	6.15	41	34.85	3	2.4	13	11.05
>20–30%	1	9.75	38	29.25	2	2	6	6
>30–40%	1	12.25	34	22.75	0	0.7	2	1.3
>40–50%	0	12.15	27	14.85	0	0.45	1	0.55
>50–60%	2	8.8	14	7.2	0	0.55	1	0.45
>60–70%	1	7.8	11	4.2	1	1.95	2	1.25
>70–80%	4	16.5	18	5.5	0	0	0	0
>80–90%	6	13.6	10	2.4	0	0	0	0
>90–100%	0	0.95	1	0.05	3	3.8	1	0.2
Total	15	89.05	216	141.95	15	21.8	216	209.2

*P-SAPS II strongly overestimates the number of deaths. Whereas PIM3 shows a quiet good fitting. O, Observed; E, Expected*.

### Repeatability of p-SAPS II

To proof the accusation of repeatability, p-SAPS II was assessed multiple times throughout the study period. The AUROCs were established and compared to the specific AUROC values of PIM3, assessed at admission, of the patients still at the PICU at the given time period. Additionally, a goodness of fit test was performed to test whether the calibration of the tests improved over the clinical stay (Table 4).

**Table 4:** The table shows the daily p-SAPS II evaluation compared to the admission PIM 3 of the patients still in the study at the given timepoint.

**Day**	***N***	**AUROC p-SAPS II (95% CI, p)**	**AUROC PIM 3(95% CI, p)**	**Difference *(p)***	**Goodness of fit p-SAPS II (p)**	**Observed/expected (Ratio)**
2	235	0.88 (0.83–0.93, *p < * 0.001)	0.716 (0.515–0.0917, *p =* 0.012)	0.164 (0.095)	13.42 (0.004)	12/32.5 (0.369)
3	234	0.86 (0.78–0.94, *p < * 0.001	0.704 (0.49–0.92, *p =* 0.023)	0.156 (0.15)	32.92 (<0.001)	11/52.15 (0.211)
7	129	0.895 (0.82–0.97, *p < * 0.001)	0.729 (0.51–0.95, *p =* 0.114)	0.165 (0.168)	8.74 (0.068)	9/21.95 (0.41)
14	51	0.857 (0.75–0.97, *p =* 0.002)	0.698 (0.45–0.94, *p =* 0.125)	0.159 (0.168)	3.15 (0.53)	8/14.2 (0.563)
21	27	0.889 (0.76–1, *p =* 0.003)	0.714 (0.44–0.99, *p =* 0.102)	0.175 (0.288)	1.82 (0.612)	7/6.65 (1.05)
28	17	0.96 (0.86–1, *p =* 0.005)	0.64 (0.29–0.99, *P =* 0.391)	0.32 (0.131)	0.296 (0.961)	5/4.25 (1.18)

*Throughout the clinical stay, the AUROC value increases although significant differences to PIM 3 were not detected. The goodness of fit test shows improvement of fitting over time. With O/E ratios adjusting to One*.

### The Calculated Mortality Risks

**Figure 2:**
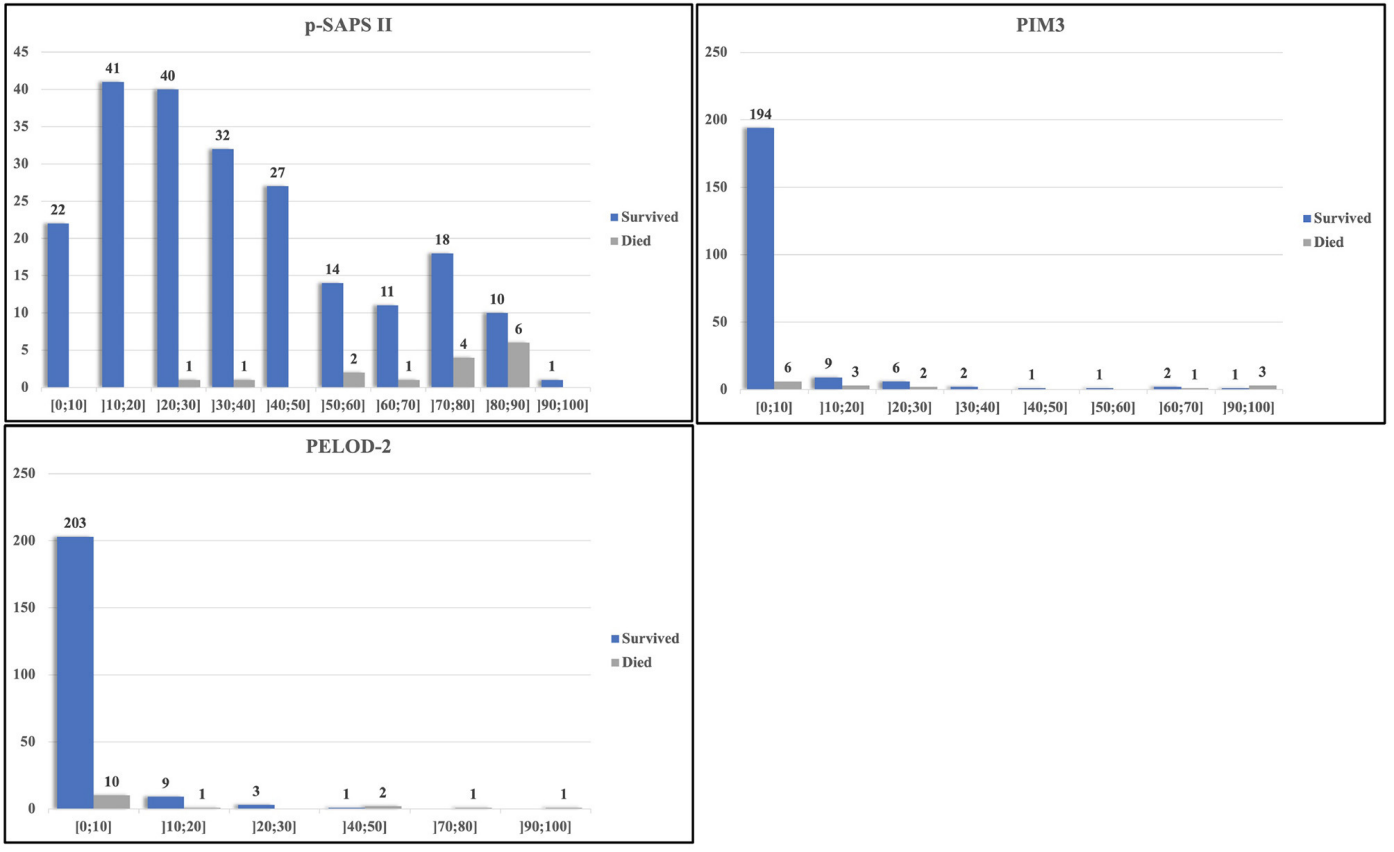
Distribution of scoring results and observed mortality.

Figure 2 depicts the calculated mortality risk distributions of survivors compared to non-survivors. PIM3 scored the largest percentage of the study population within the first risk group; 86% (199/231) of the admissions were scored within the 0–10% risk group. In the p-SAPS II group the study population revealed a wider distribution.

**Figure 3:**
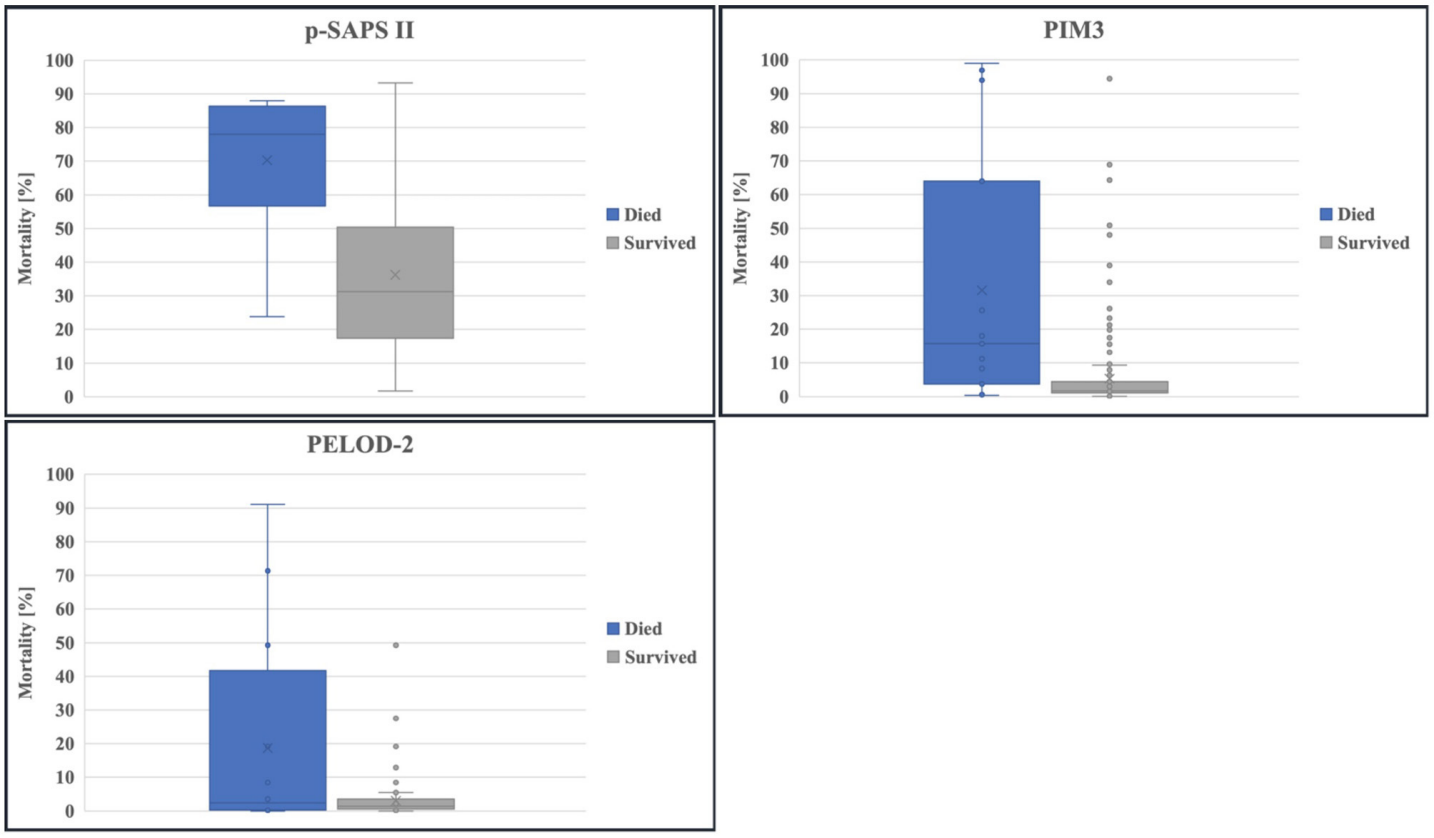
Boxplot of calculated mortality risks: survivors compared to non-survivors.

The calculated mortality risk resulting from p-SAPS II of non-survivors shows a significantly higher value compared to survivors as seen in Figure 3: (70.3/ 95% CI 60.11–79.3 vs. 36.29/ 95% CI 33.21–39.27, *p* < 0.0001). The same applies for PIM3 (31.57/ 95% CI 14.04–50.43 vs. 5.33/ 95% CI 2.38–3.83, *p* = 0.001). PELOD-2 reveals no significant differences (19.95/ 95% CI 5.77–35.45 vs. 3.02/ 95% CI 2.38–3.83, *p* = 0.231).

### Logistic Regression of p-SAPS II Combined With PIM3 to Mortality

The logistic regression of p-SAPS II combined with PIM3 to mortality revealed that in a model with an already-known p-SAPS II, the additional calculation of PIM3 had no significant effect on the accuracy of predicting mortality (*p* = 0.062). In contrast, the additional calculation of p-SAPS II increased the accuracy of the calculated risk of mortality, but only when PIM3 was known (*p* = 0.003).

### Model Optimization

The p-SAPS II was strongly modeled on the original expanded SAPS II. Most of the categories were directly overtaken or slightly adjusted according to age and gender. Our modified classification “main pediatric diagnoses” (Supplementary Material Table 2) also indicates whether the admission was scheduled or unscheduled. In order to simplify the score and make assessment easier, variables “age,” “sex,” and “type of admission” were excluded as they were deemed redundant.

The calculated AUC for this optimized model was 0.90 (CI: 0.84–0.96; *p* < 0.001), the performed goodness-of-fit test revealed a good calibration model (*p* = 0.098).

A second alternative version of p-SAPS II calculated without the additional variable of “clinical category” reflected notable results: AUROC = 0.87 (CI: 0.79–0.95; *p* < 0.001); goodness-of-fit test: *p* = 0.435.

When comparing these new variants of the p-SAPS II with the “gold standard” PIM3, and our newly-created p-SAPS II, the new, optimized versions reflected a higher discrimination potential and had well-performing goodness-of-fit tests, despite the differences not being statistically significant.

## Discussion

### Decision for the Expanded SAPS II as the Score of Origin

In the course of searching for an appropriate scoring system for pediatric intensive care patients, the newly-created p-SAPS II was compared to PELOD-2, pSOFA and PIM3. We decided to adapt the expanded SAPS II instead of SAPS II or SAPS 3 because it has been shown that SAPS II likely overpredicts mortality risk. For example, Metnitz et al. performed a multicenter study in 1999 (18) including 1,733 patients. According to their results, the calibration of SAPS II was poor (*p* < 0.001).

In 2005 an expanded version of SAPS II (expanded SAPS II) was published by Gall et al. (11), where SAPS II was updated based on data from over 100,000 consecutive first-time admissions. This study provided valuable information concerning an excellent calibration of expanded SAPS II (Hosmer-Lemeshow test: *p* = 0.81).

Several studies have been carried out to investigate the goodness-of-fit of SAPS 3. In the framework of a systematic review of 28 external validation studies by Nassar et al. it was shown that SAPS 3 required individual, clinic-specific customization before successfully applying it in clinical routines due to the poor calibration of the standard version (15). The expanded SAPS II usually provides more accurate results without specific modification.

### p-SAPS II Shows Excellent Discrimination Potential Analyzed Through AUROC, but Lacks Predictive Accuracy

In our analysis, the ROC curves illustrate the validity of every score concerning the discrimination potential between survivors and non-survivors, according to the scored points/ calculated mortality risks independent of the reason of admission (19).

In the framework of our study, p-SAPS II performed significantly better than PELOD-2 and pSOFA in terms of discrimination between survivors and non-survivors.

Although a trend for improved performance of p-SAPS II compared to the PIM3 was visible, differences between the AUROCs were not statistically significant.

Slater et al. found that PIM3 showed an excellent discrimination (survivor/ non-survivor: AUROC = 0.9068) and good calibration (Hosmer-Lemeshow-Goodness-of-fit test: χ^2^ *p* > 0.1) (20); these are somewhat better than our PIM3 results. The poor fit of diagnoses of our study population compared to those being part of PIM3 may be a reason for the different AUROCs.

To our knowledge, there are no published versions of expanded SAPS II for predicting mortality in children. Le Gall et al. performed internal and external validation studies in the framework of expanded SAPS II for adults (11). Their results concerning the discrimination values are similar to ours. The mean AUROCs of expanded SAPS II were 0.879 and 0.8787 by using an additional external validation dataset from an external population; the AUROC of our p-SAPS II was 0.86. The differences within the AUROCs of the latter scores were most visible in areas with a high sensitivity. In these areas, low threshold points were chosen to discriminate between possible survivors and non-survivors to effectively extract those having the highest probability of dying (Figure 1).

In our PIM3 scores, around 87% of the admissions and more than 40% of all non-survivors were scored in the 0–10% mortality risk group (Figure 2). Due to this poor distribution, many survivors scored higher or comparable to the non-survivors, leading to false positives at the high sensitivity area in the PIM3 AUROC. The distribution of points in p-SAPS II is more spread out and thus gives a more coherent picture of the patient collective. Since the first non-survivor appears in the risk group of 20–30% mortality risk, the number of false positive patients, using low thresholds with high sensitivity in the AUROC, was lower, resulting in higher AUROC values. Although the differences of the AUROCs were not significant, p-SAPS II tended to perform better, especially in children having a low calculated mortality score when using PIM3 (Figure 2).

Nonetheless, the total prediction of mortality risk was highly accurate for PIM3, whereas the prediction accuracy was low for the p-SAPS II.

A more detailed comparison between the two scores within our data could be seen through linear regression analysis. This means, that additional assessment of p-SAPS II was able to add value and information to an existing PIM 3 in terms of mortality prediction. It was concluded that p-SAPS II, although its predictive value is limited due to missing calibration, predicts mortality with high accuracy, due to its excellent discrimination potential. p-SAPS II can effectively assess critical clinical situations with an increased mortality risk. The potential pitfall of this score so far is the calculation of mortality from the assessed p-SAPS II points at admission. Repeated scoring although increased mortality prediction accuracy as can be seen in Table 4. When comparing the distribution of patients within the mortality risk groups, the calculated mortality risks are far more spread out within different categories of mortality than in the PIM3. It could be argued that p-SAPS II could classify and describe the patient's situation in a more detailed manner than binary “yes” or “no”.

While PIM3 gave accurate mortality predictions, p-SAPS II reflected the general severity of a patient's clinical state more accurately, as shown in the linear regression model.

In contrast to the predictive score PIM3, which is designed to be performed just one single time at admission and should not be repeated. By definition, p-SAPS II is designed as a descriptive score, and can be performed more often throughout the patients stay at the PICU exactly as the score it originated from, the expanded SAPS II, being a considerable advantage as it can easily be applied to continually follow the patient's course through the entire clinical stay. Table 4 proofed the repeatability of the score and showed that following a patient over time improved accuracy of mortality prediction. Of course, it has to be stated that this table is somehow biased as only the sickest and most instable patients stayed at the PICU for longer than 21 days while all others were transferred. Therefore, the low number of patients and the composition of patients in the latest assessment groups may limit the statistical relevance.

### Limitations of the Study

#### P-SAPS II Overestimates the Mortality Risk

The predictive capacity of p-SAPS II for clinical use was a large feature of our study. The actual mortality risk was overestimated at admission day, using the original formula from the expanded SAPS II, a finding also reported by Sathe et al. (21) for the expanded SAPS II.

While mortality prediction formulas are complex calculations derived as the result of long calibration processes, they are, however, inflexible. In light of ever-improving treatment options for critical clinical situations that result in decreased mortality rates despite high risk scores, mortality prediction formulas can become outdated rather quickly. Another shortfall is that we changed the expanded SAPS II to the p-SAPS II but did not change the mortality risk formula. To adequately fit within the framework of our scoring model, the formula should be adjusted within a calibration study. However, our study population was too small to perform an adequate calibration, which was not, in any case, within the scope of this proof of principle study. But it has to be considered that repeating the scoring improved prediction accuracy (Table 4).

Interestingly, the optimized models of p-SAPS II revealed a better fit when using the formula from the original expanded SAPS II. In general, the optimized p-SAPS II versions performed better in terms of distribution capacity, although the differences were not statistically significant.

The reason behind the improved AUROCs of the optimized models could be that the influence of the “age” variable and distribution of the given points were only assumptions, possibly producing misleading results. Contrary to the original expanded SAPS II, the effects of age and gender were already considered in the percentile curves of the numeric variables; therefore, one uncertainty factor could be removed from the score.

#### Small Population Size

It should also be taken into consideration that our study population derived from one single center, when comparing our data with other multicenter studies.

For example, a total of 117 unit-years (total number of units = 60) of data were used for model-building and validating PIM3. A total of 53.112 admissions were used for model building (5). Given this discrepancy it is obvious that our study can solely be seen as a proof of principle study and further research with bigger data sets has to be performed before general statements about usability and applicability can be done. Due to the small sample size validation processes were not performed as discussed below.

#### Missing Data

Due to the retrospective character of the study, one limitation was missing data, particularly in the case of calculated Glasgow coma scale (GCS) and PaO_2_ values. It should be noted that about one-half of PICU stays were patients recovering from surgical procedures. In the majority of these cases these patients were sedated and intubated upon admission. GCS should therefore be recorded before sedation to accurately assess a patient's awareness. In our study, GCS was assumed to be normal; thus, these patients were not assigned points for the “awareness” category. The problem of missing values was addressed by Slater et al. (20), This study states that “missing” values like the PaO_2_ most often were not actually missing values, but were not recorded from the patient due to lack of necessity in clinical routine, when the patient's condition was in no need for this diagnostic criteria (20). Calculation of PaO_2_ did not increase performance of PIM 3. As in the biggest part of the cases the “missing” values did not lead to pathological results. Therefore, most of these values were not pathological and it can be argued, that values that are not stated pathological can be assumed as normal.

#### General Validity and Validity Testing

As many of the diagnoses at our PICU, were not applicable for the original PIM3 classification system, we adapted this variable for our PICU collective as described in the methods section above. For use in the p-SAPS II. Considering the large data volume used to establish PIM3 and its good calibration (20), we adopted the PIM3 classification method, and expanded it with diagnoses and reasons for admissions recurrent in our PICU (Supplementary Material Table 2).

It needs to be emphasized that the study was performed at a PICU that is part of the national heart center, therefore most of our patients suffered from cardiac diagnoses (Table 2).

This may limit the overall comparability of our study population to other PICUs.

Another limitation can probably be seen in the diagnoses that were implemented in the new variable “main pediatric diagnosis” (Supplementary Material Table 2). It has been tried to add general applicable diagnoses from the PICU population. Over-fitting of our score to the specific study population cannot be ruled out. Due to the small sample size and the proof of principle character of the study no validation processes were performed so far. These steps have to be performed in a bigger approach including calibration and validation processes with external data sets.

#### Usability

Most of the variables needed for the calculation of the p-SAPS II were part of routine monitoring in the PICU. Additionally, they were recorded electronically in our PICU whereby performing the score was facilitated. Furthermore, the calculations can be eased using a calculator tool already available as a prototype. No specific assessment of burden to take the different scores were performed.

## Conclusion

Our study revealed that the newly-created p-SAPS II performed better than the established PIM3 in terms of discriminating between survivors and non-survivors. A particularly advantageous feature of our p-SAPS II is the fact that it was designed and developed to be performed on a regular basis throughout the PICU stay. Our score also had the highest AUROCs of all those tested, although the differences were not always significant. There is a need to further optimize calibration and formulas of our p-SAPS II to predict mortality more accurately as mortality rates were overestimated at admission in our study population. Repeating the score over time did not improve discrimination potential but increased mortality prediction accuracy. To simplify the calculation and integrate the score in routine, a calculation tool can be helpful, which is already under development and usable as a prototype.

Our study provides compelling reasons for establishing a pediatric scoring model. By future studies we aim to optimize the predictive value and to show the general usability of this promising scoring method for a broad use in PICUs all over the world.

## Data Availability Statement

The raw data supporting the conclusions of this article will be made available by the authors, without undue reservation.

## Ethics Statement

The studies involving human participants were reviewed and approved by Ethikkommission der Medizinischen Universität Wien (Borschkegasse 8b/E06, 1090 Wien). Written informed consent from the participants' legal guardian/next of kin was not required to participate in this study in accordance with the national legislation and the institutional requirements.

## Author Contributions

SI performed the analysis of data and writing of the manuscript. JV participated in the design and execution of the study, and performed data collection, statistical analysis and interpretation of the data. JG supervised the study and is the program director and head of the PICU. GS helped with extraction and interpretation of the data. JB performed interpretation of the data and critical editing of the manuscript. CK performed statistical advice and interpretation. MH conceived the study and participated in its design and execution, the analysis of data and writing of the manuscript. MH had full access to all data in the study and takes responsibility for the integrity of the data and accuracy of the data analysis. All authors read and approved the final manuscript.

## Conflict of Interest

The authors declare that the research was conducted in the absence of any commercial or financial relationships that could be construed as a potential conflict of interest.

## Publisher's Note

All claims expressed in this article are solely those of the authors and do not necessarily represent those of their affiliated organizations, or those of the publisher, the editors and the reviewers. Any product that may be evaluated in this article, or claim that may be made by its manufacturer, is not guaranteed or endorsed by the publisher.

## Abbreviations

p-SAPS II: age-adapted version of the expanded Simplified Acute Physiology Score II/ pediatric SAPS II
AUROC: area under the receiver operating characteristic curve
expanded SAPS II: expanded Simplified Acute Physiology Score II
GCS: Glasgow Coma Scale
GOF: Goodness-of-Fit test
ICU: Intensive Care Unit
PIM3: Pediatric Index of Mortality 3
PICU: Pediatric Intensive Care Unit
PELOD-2: Pediatric Logistic Organ Dysfunction Score-2
pSOFA: Pediatric Sequential Organ Failure Assessment Score
O/E ratio: Ratio of “observed&#8221
against “expected&#8221
deaths
ROC: Receiver operation characteristic
SAPS 3: Simplified Acute Physiology Score 3.
