# Supplementary material for: Pediatric Simplified Acute Physiology Score II: Establishment of a New, Repeatable Pediatric Mortality Risk Assessment Score
Source: Front Pediatr. 2021 Oct 28;9:757822. doi: 10.3389/fped.2021.757822 (PMC8583491; doi:10.3389/fped.2021.757822)
Supplement: Supplementary file 1 [file Table_1.docx]

## Supplementary Material

| **Variable** | **Age** |  | | | | | | | | | | | | | | | | | | | | |  |
| --- | --- | --- | --- | --- | --- | --- | --- | --- | --- | --- | --- | --- | --- | --- | --- | --- | --- | --- | --- | --- | --- | --- | --- |
| **Heart rate (per minute) (22)** |  | **11** | | | | **2** | | | | | | | **0** | | | | **4** | | | | | **7** | **Points** |
|  | 0-<3mo  3-<6mo  6-<9mo  9-<12mo  12-<18mo  18-<24mo  2-<3y  3-<4y  4-<6y  6-<8y  8-<12y  12-<15y  15-<18y | <104  <99  <95  <92  <88  <83  <78  <72  <67  <62  <57  <52  <49 | | | | 104-118  99-113  95-109  92-106  88-102  83-97  78-92  72-87  67-82  62-76  57-71  52-65  49-61 | | | | | | | 119-164  114-159  110-156  107-153  103-149  98-146  93-142  88-138  83-134  77-128  72-120  66-112  62-107 | | | | 165-185  160-181  157-177  154-175  150-172  147-169  143-166  139-163  135-160  129-154  121-146  113-137  108-131 | | | | | >185  >181  >177  >175  >172  >169  >166  >163  >160  >154  >146  >137  >131 | Values |
| **Systolic blood pressure (mmHq) (2, 23)** |  | **13** | | **5** | | | | **0** | | | **2** | | | | | | | | | | | | **Points** |
|  | 0-<1mo  1 mo-<1y  1–12y  >12y | <35  <35  <45  <55 | | 35-65  35-75  45-85  55-95 | | | | >65  >75  >85  >95 | | |  | | | | Boys/Girls | | | | |  | Boys/Girls | | Values |
|  |  |  |  |  |  |  |  |  |  |  | 1y  2y  3y  4y  5y  6y  7y  8y | | | | >99/ >100  >102/ >101  >105/ >103  >107/ >104  >108/ >106  >110/ >108  >111/ >109  >112/ >111 | | | | | 9y  10y  11y  12y  13y  14y  15y  16y  17y | >114/ >113  >115/ >115  >117/ >117  >120/ >119  >122/ >121  >125/ >122  >127/ >123  >130/ >124  >132/ >125 | |  |
| **SpO_2_/FiO_2_ (9, 24, 25)** |  | **11** | | | | | | | **9** | | | | | | | | | **6** | | | | **0** | **Points** |
|  |  | <148  With respiratory support | | | | | | | 148-220  With respiratory support or not | | | | | | | | | 221-291 | | | | ≥292 | Values |
| **Sodium (mEq/L) (26)** |  | **5** | | | | | | | | | **0** | | | | | | | | **1** | | | | **Points** |
|  | 0-11mo  ≥1y | <133  <136 | | | | | | | | | 133-142  136-145 | | | | | | | | >142  >145 | | | | Values |
| **Potassium (mEq/L) (26)** |  | **3** | | | | | | | | | **0** | | | | | | | | **3** | | | | **Points** |
|  | ≤2mo  3-11mo  ≥1y | <4.0  <3.7  <3.5 | | | | | | | | | 4.0-6.2  3.7-5.6  3.5-5.5 | | | | | | | | >6.2  >5.6  >5.5 | | | | Values |
| **Bilirubin (mg/dL) (9)** |  | **0** | | | | | | | | | **4** | | | | | | | | **9** | | | | **Points** |
|  |  | <1.2 | | | | | | | | | 1.2-5.9 | | | | | | | | >5.9 | | | | Values |
| **Type of admission (12)** |  | **o** | | | | | | | | | **6** | | | | | | | | **8** | | | | **Points** |
|  |  | Scheduled surgical | | | | | | | | | Medical | | | | | | | | Unscheduled surgical | | | | Values |
| **Risk**  **Diagnoses**  **(Supplementary Table 2)** |  | **2** | | | | | | | | **8** | | | | | | | | | | **29** | | | **Points** |
|  |  | Low-risk | | | | | | | | High-risk | | | | | | | | | | Very high-risk | | | Values |
| **Age** |  | **7** | | | | | **12** | | | | | | | **15** | | | **16** | | | | **18** | | **Points** |
|  |  | 144-216 mo | | | | | 72-143 mo | | | | | | | 12-71 mo | | | 1-11 mo | | | | 0-30 days | | Values |
| **GCS (27)** |  | **26** | | | **13** | | | | | | | **7** | | | | **5** | | | | | **0** | | **Points** |
|  |  | <6 | | | 6-8 | | | | | | | 9-11 | | | | 11-13 | | | | | 14-15 | | Values |
| **WBC (10^3^/mm^3^)**  **(10, 26)** |  | **12** | | | | | | | | | **0** | | | | | | | | | **3** | | | **Points** |
|  | ≤1mo  2-11mo  1-6y  7-12y  13-18y | <2  <2  <2  <2  <2 | | | | | | | | | 2 (5)-19.5  2 (6)-17.5  2 (5)-14.5  2 (5)-14.5  2 (4.5)-13.5 | | | | | | | | | >19.5  >17.5  >14.5  >14.5  >13.5 | | | Values |
| **BUN (mg/dl)**  **(26, 28)** |  | **0** | | | | | | | | | **6** | | | | | | | | | **10** | | | **Points** |
|  | 0-1y  2-15y  16-18y | 8-28  5-25  5-20 | | | | | | | | | >28-51  >25-68  >20-68 | | | | | | | | | >51  >68  > 68 | | | Values |
| **Urinary output (ml/kg/hr) (29)** |  | **11** | | | | | | | | | **4** | | | | | | | | | **0** | | | **Points** |
|  |  | No urine, Anuria | | | | | | | | | <1.0 ml/kg/hr, Oliguria | | | | | | | | | 1-2 ml/kg/hr | | | Values |
| **Temperature (°C)**  **(12, 30)** |  | **Measurement method** | | | | | | | | | **0** | | | | | | | | | **3** | | | **Points** |
|  |  | Rectal  Ear, oral, axillary/forehead | | | | | | | | | <38.5°C  <38.0°C | | | | | | | | | ≥38.5°C  ≥38.0°C | | | Values |
| **Serum bicarbonate (mEqu/L)  Boys (31, 32)** |  | **6** | | | | | | | | | **3** | | | | | | | | | **0** | | | **Points** |
|  | 1-<3y  3-<6y  6-<9y  9-<12y  12-<15y  15-<19y | <15  <15  <15  <15  <15  <15 | | | | | | | | | 15-16  15-17  15-18  15-19  15-20  15-21 | | | | | | | | | 17-24  18-25  19-26  20-27  21-28/29  22-29 | | | Values |
| **Serum bicarbonate**  **(mEqu/L) Girls (31, 32)** |  | | **6** | | | | | | | | **3** | | | | | | | | | **0** | | | **Points** |
|  | 1-<3y  3-<6y  6-<9y  9-<12y  12-<15y  15-<19y | | <15  <15  <15  <15  <15  <15 | | | | | | | | 15-17  15-17  15-18  15-19  15-20  15-20 | | | | | | | | | 18-24  18-25  19-26  20-27  21-28  21-28 | | | Values |
| **Expanded SAPS II (age-adapted) / p-SAPS II (11)** |  | | | Original SAPS II score | | | | | | | | | | | | | | | | 0.0742 × SAPS II | | | |
|  | **Age** | | | 12–18 years  6–12 years  12–60 months  1–11 months  0-30 days | | | | | | | | | | | | | | | | 0  0.1639  0.2739  0.3690  0.6645 | | | **Points** |
|  | **Sex** | | | Male  Female | | | | | | | | | | | | | | | | 0.2083  0 | | |  |
|  | **Length of hospital stay before ICU admission** | | | <24 hours 1 day 2 days 3–9 days >9 days | | | | | | | | | | | | | | | | 0 0.0986 0.1944 0.5284 0.9323 | | |  |
|  | **Patient's location before ICU** | | | Emergency room or mobile emergency unit Ward in same hospital Other hospital | | | | | | | | | | | | | | | | 0  0.2606 0.3381 | | |  |
|  | **Clinical category** | | | Medical patient Other | | | | | | | | | | | | | | | | 0.6555 0 | | |  |
|  | **Intoxication** | | | No Yes | | | | | | | | | | | | | | | | 1.6693 0 | | |  |

Table*1*: The newly created p-SAPS II with all age adapted parameters

Abbreviations: Mo=months, y=years, SpO_2_=oxygen saturation, FiO_2_=fraction of inspired oxygen, GCS=Glasgow Coma Scale, WBC=white blood cells, BUN=blood urea nitrogen

SI conversion factors: To convert Bilirubin to µmol/L, multiply values by 17.1, to convert BUN to mmol/L, multiply values by 0.357.

Instruction: Always record the worst values. Choose the appropriate points according to all variable thresholds. Values that are unavailable correspond to 0 points. Add up all points. Then, multiply the points by the factor of 0.0742. Then create a total sum of the following coefficients relating to the categories of “Expanded SAPS II (age-adapted) / p-SAPS II”: age, sex, length of hospital stay before ICU admission, patient’s location before ICU, clinical category and intoxication. Finally, add the calculated sum to the first result (SAPS II, points multiplied by the factor 0.0742) and use the following formulas:

Logit = -14.4761 + 0.0844 x score + 6.6158 x (score + 1).

Risk of mortality = exp(logit) / (1+exp [logit])
