# Supplementary material for: Pediatric Simplified Acute Physiology Score II: Establishment of a New, Repeatable Pediatric Mortality Risk Assessment Score
Source: Front Pediatr. 2021 Oct 28;9:757822. doi: 10.3389/fped.2021.757822 (PMC8583491; doi:10.3389/fped.2021.757822)
Supplement: Supplementary file 2 [file Table_2.docx]

## Supplementary Material

|  | **Low-risk**  Mortality risk <4/5 %  OR <1 | **High-risk**  Mortality risk 4/5-20/30%  OR 1-5 | **Very high-risk**  Mortality risk >20/30%  OR >5 |
| --- | --- | --- | --- |
| **Respiratory disease** | - Asthma (5) - Bronchiolitis/(viral) Pneumonia (5) - Croup (5) - Obstructive sleep apnea (5) - Mild ARDS (33) | - Moderate ARDS (33) | - Severe ARDS (33) |
|  |  | - Pulmonary hypertension (34, 35) |  |
| **Cardiovascular disease** | - STS-EACTS mortality category 1 (36) - STS-EACTS mortality category 2 (36) | - STS-EACTS mortality category 3 (36) - STS-EACTS mortality category 4 (36) - Cardiomyopathy - Myocarditis (5) - Hypoplastic left heart syndrome (5) | - STS-EACTS mortality category 5 (36) - Cardiac arrest/CPR/shock:   cardiogenic, obstructive, hypovolemic (5, 37) |
| **Miscellaneous** | - Diabetic ketoacidosis (5) | - Inborn errors of metabolism (38) | - Mitochondrial diseases,   trisomy 18, trisomy 13 (39, 40) |
| **Neurologic diseases** | - Seizure disorder (5) - Acute Neuromuscular Disorders**^a^** (41) | - ICH score=1, ICH score=2 (42) - Spontaneous cerebral hemorrhage (if ICH score is not possible) (5) - Encephalitis (43) - Neurodegenerative disorder (5) | - ICH score ≥3 (42) |
| **Recovery from neurosurgical procedures** | - Craniotomy/ Craniectomy: if preoperative seizure history**^b^** (44) - Defect repair: CNS structural abnormality**^b^** (44) - Shunts/Implants: cerebral palsy**^b^** (44) | - Craniotomy/ Craniectomy: development delay, IVH grade 3 or 4, chemotherapy**^b^** (44) - Laminectomy: development delay**^b^** (44) - Shunts/Implants: CNS tumor**^b^** (44) | - Craniotomy/ Craniectomy: age <1 year**^b^** (44) |
| **Hepatic disease** |  |  | - Liver failure (5) |
|  |  | - Sepsis (45) | - Septic shock, distributive shock (45-47) |
| **Renal disease** |  |  | - Acute kidney failure (48) |
| **Immune-related disease**  **Hematological disease** |  |  | - Severe combined immune deficiency (5) - Leukemia or lymphoma after first induction (5) - Bone marrow transplant recipient (5) |
| **Injuries** | - Burn injuries (TBSA <20%) (49) | - Burn injuries (TBSA 20-40%) (49) - Severe traumatic brain injury (with metabolic and respiratory acidosis, or respiratory alkalosis) (50, 51) | - Burn injuries (TBSA >40%) (49) |

Table*2*: p-SAPS II: Supplemented diagnoses and reasons for admission based on PIM3 classification *(5)* Abbreviations: TBSA = Total body surface area, ARDS = Acute Respiratory Distress Syndrome, STS-EACTS = Society of Thoracic Surgeons - European Association for Cardio-Thoracic Surgery, CPR = cardiopulmonary resuscitation, ICH = Intracerebral hemorrhage, IVH = intraventricular haemorrhage, ^a^Acute Neuromuscular Disorders: Disorders of the Anterior Horn Cell, Disorder of the Nerve Root and Peripheral Nerves, Chronic Inflammatory Demyelinating Polyneuropathy, Disorders of the Neuromuscular Junction, Disorders of Muscle, ^b^risk factors

Supplemented diagnoses and reasons for admission based on PIM3 classification

- *Acute Respiratory Distress Syndrome (ARDS):*

In this study the definition of ARDS and the recommendations from the Pediatric Acute Lung Injury Consensus Conference were implemented because it is shown that differences exist between ARDS in children and ARDS in adults (33). Moreover, it was also taken into account that in PICUs measurements from noninvasive monitoring are usually more prevalent than those from invasive monitoring (33).

- *Society of Thoracic Surgeons (STS) – European Association for Cardiothoracic Surgery (EACTS) mortality category 1-5*

Because there are many admissions for the purpose of recovering from cardiac procedures, the classification published by O´Brien et al was used for identifying at-risk children undergoing cardiac surgery (36).

- *Acute Neuromuscular Disorders*

There are many diseases that fall within this group. Using the results of Harrar et al, in this study ‘Acute Neuromuscular Disorder’ was certified as a low-risk diagnosis. This specifically relates to disorders of the: anterior horn cell; nerve root and peripheral nerves; neuromuscular junctions; and disorders of the muscle (41).

- *Recovery from neurosurgical procedures*

Children with neuro-oncological or neurological diseases are admitted to the PICU after neurosurgical procedures. For this reason, it was necessary to identify increased perioperative mortality risk and the odds ratios were integrated for different neurosurgical procedures as calculated by Kuo et al (44).

- *Burn injuries*

Considering that burn injuries can cause multiple organ dysfunction syndrome (49), it was decided to classify burn injuries by total body surface area; as done by Dewi et al (49).

- *Pulmonary hypertension*

When mean pulmonary artery pressure is higher than 25 mmHg, it is classified as pulmonary hypertension. Because of the high death rate, pulmonary hypertension was considered a high-risk diagnosis (34, 35).

- *Inborn errors of metabolism (IEM)*

Nowadays inborn errors of metabolism can be detected during newborn screening and are treatable when recognized at an early stage. Under certain circumstances, however, if other organ dysfunction is present, inborn errors of metabolism may result in severe consequences (38).

- *Intracerebral Hemorrhage Score (ICH)*

The Pediatric ICH was considered appropriate to evaluate an intracerebral hemorrhage because many relevant symptoms or consequences are considered when utilizing this score (42).

- *Encephalitis*

On the basis of the mortality rate or relative risk as published by Rao et al (43), encephalitis was regarded as a high-risk diagnosis.

- *Sepsis*

Considering the mortality rate of six percent (45) and possibility of septic shock, sepsis was regarded as a high-risk diagnosis.

- *Severe traumatic brain injury*

Referring to the findings of Rahimi et al (50), the highest odds ratios (3.94; p=0.012) were calculated in the events of traumatic brain injury with metabolic acidosis and respiratory acidosis at the time of admission. Moreover, Reisner et al reported a death rate ranging from 19 to 32 percent (51); for this reason, (severe) traumatic brain injury was deemed a high-risk diagnosis.

- *Shock: cardiogenic, obstructive, hypovolemic, distributive*

The most common cause of shock in the Vienna General Hospital’s PICU is distributive shock; more precisely, septic shock. According to different studies, shock has been rated as a very high-risk diagnosis (37, 45-47).

- *Mitochondrial diseases, trisomy 13 and 18*

These disorders are associated with a high risk of mortality within the first months/years of life (85, 86).

- *Acute kidney failure*

The retrospective data analysis performed by Williams et al (48) illustrated the high mortality risk in children with acute kidney injury; for this reason this disorder was classified as a very high-risk diagnosis.
